# Control of high-speed jumps in muscle and spring actuated systems: a comparative study of take-off energetics in bush-crickets (*Mecopoda elongata*) and locusts (*Schistocerca gregaria*)

**DOI:** 10.1007/s00360-023-01524-2

**Published:** 2023-10-19

**Authors:** Chloe K. Goode, Charlie Woodrow, Shannon L. Harrison, D. Charles Deeming, Gregory P. Sutton

**Affiliations:** 1 School of Life and Environmental Sciences, University of Lincoln, Joseph Banks Laboratories, Green Lane, Lincoln, LN6 7DL UK; 2 Department of Ecology and Genetics, Evolutionary Biology Centre, Uppsala University, Norbyvägen 18 D, 752 36 Uppsala, Sweden

**Keywords:** MA, LaMSA, Pitch, Orthoptera, Biomechanics

## Abstract

The Orthoptera are a diverse insect order well known for their locomotive capabilities. To jump, the bush-cricket uses a muscle actuated (MA) system in which leg extension is actuated by contraction of the femoral muscles of the hind legs. In comparison, the locust uses a latch mediated spring actuated (LaMSA) system, in which leg extension is actuated by the recoil of spring-like structure in the femur. The aim of this study was to describe the jumping kinematics of *Mecopoda elongata* (Tettigoniidae) and compare this to existing data in *Schistocerca gregaria* (Acrididae), to determine differences in control of rotation during take-off between similarly sized MA and LaMSA jumpers. 269 jumps from 67 individuals of *M. elongata* with masses from 0.014 g to 3.01 g were recorded with a high-speed camera setup. In *M. elongata*, linear velocity increased with mass^0.18^ and the angular velocity (pitch) decreased with mass^−0.13^. In *S. gregaria*, linear velocity is constant and angular velocity decreases with mass^−0.24^. Despite these differences in velocity scaling, the ratio of translational kinetic energy to rotational kinetic energy was similar for both species. On average, the energy distribution of *M. elongata* was distributed 98.8% to translational kinetic energy and 1.2% to rotational kinetic energy, whilst in *S. gregaria* it is 98.7% and 1.3%, respectively. This energy distribution was independent of size for both species. Despite having two different jump actuation mechanisms, the ratio of translational and rotational kinetic energy formed during take-off is fixed across these distantly related orthopterans.

**Supplementary Information:**

The online version contains supplementary material available at 10.1007/s00360-023-01524-2.

## Introduction

Jumping is a form of locomotion employed by a wide range of insects and is particularly well utilised by orthopteran insects, such as locusts, crickets, and bush-crickets (Bennet Clark 1975; Burrows and Morris 2003). Jumping is thought to provide the animal with a faster means of travelling than crawling (Bertone et al. 2022), is a crucial aid in predator evasion (Cofer et al. 2010; Hawlena et al. 2011; Moore et al. 2017), and can act as a prerequisite to flight (Wan et al. 2020). Executing a successful jump requires control of speed, elevation, and rotation (Sutton and Burrows 2011; Goode and Sutton 2023), and an insect’s ability to control these will depend on their mass, morphology, and actuation mechanism.

Insects can actuate their jumps through one of two pathways: using pre-loaded springs or by direct muscle actions. In latch mediated spring actuated (LaMSA) jumpers, the muscles first store mechanical energy in a latched elastic structure and subsequently unlatching of this structure allows the spring to recoil (Burrows 2010; Ribak & Weihs 2011; Longo et al. 2019; Patek 2023). In the locust, *Schistocerca gregaria* (a model organism for LaMSA system mechanics), the leg is held in place by a geometric latching system, that can be released by relaxation of the tibia flexor muscle (Heitler 1977). The spring consists of two structures in the femur: the extensor apodeme and the semi lunar process (Bennet Clark 1975; Cofer et al. 2010; Sutton et al. 2019). This system provides a take-off linear velocity that is independent of body mass (Katz and Gosline 1992; Ilton et al. 2018), and a decrease in take-off angular velocity (pitch) with body mass (Goode and Sutton 2023). The energy distribution between these two velocities is, however, fixed and formed at take-off, with a distribution of 98.7% to translational kinetic energy and 1.3% to rotational kinetic energy (Goode and Sutton 2023). Consequently, in LaMSA systems, smaller individuals spin faster during a jump, which is observed across other insects that use elastic recoil mechanisms and a general rule for smaller jumping animals (Alexander 1995; Scholz et al. 2005; Sutton et al. 2019).

Conversely, in muscle actuated (MA) systems, leg extension is caused by contraction of the extensor muscles that straightens the previously flexed leg. In these systems the take-off velocity is constrained by amount of mechanical power that can be generated by a muscle (about 100 watts per kg, Sutton et al. 2016, 2019). Larger animals with larger muscles are able to extend their legs over longer times, generating more energy and achieving higher take-off velocities (Sutton et al. 2019). Despite a good understanding of LaMSA system mechanics, few studies have yet investigated in detail how linear and angular velocity change with either mass or the translational and rotational energetics of MA systems. Comparing such processes in MA and LaMSA systems across body masses (i.e., through ontogeny) could provide detailed insights into how jumping behaviours are shaped by mass, life history, and actuation mechanism.

The jumping behaviour of LaMSA jumpers, such as *S. gregaria*, has been extensively studied regarding posturing, kinematics and energy storage involved in the jump (Burrows 2006, 2009; Burrows et al. 2008; Sutton and Burrows 2011; Baek et al. 2020; Wan et al. 2020; Goode and Sutton 2023). Comparable understanding of jumping behaviour in a similarly sized MA jumper would be beneficial for comparisons of the two systems. To address this, we here investigate jumping mechanics in the bush-cricket *Mecopoda elongata*, which displays a similar size range to its orthopteroid cousin *S. gregaria*. Unlike grasshoppers, bush-crickets are currently only known to jump using muscle actuated systems (Burrows and Morris 2003). We measured kinematics of jumping *M. elongata* across a range of body masses from 0.014 g to 3.01 g, which represent the first instar through to the adult, and assessed linear and angular (pitch) take-off velocity. We used this information to determine the distribution of translational and rotational kinetic energy in the energy distribution of the bush-cricket. Finally, we compare the results to previously published mirrored data in the locust.

## Methodology

### Housing

**Fig. 1:**
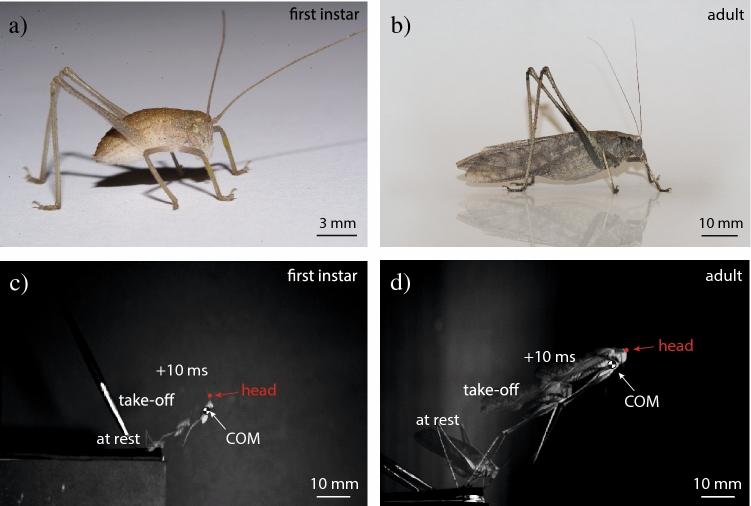
The bush-cricket used in the study (*Mecopoda elongata*) and jumping data methodology. **a** Picture of a first instar nymph. **b** Picture of an adult male. **c** First instar nymph example jump progression used in analysis. **d** Adult example jump progression used in analysis. *COM* = centre of mass

Sixty-seven captive bred bush-crickets *Mecopoda elongata* (Linneaus 1758) ranging in body mass (BM in g) from 0.014 g to 3.01 g (Fig. 1a, b) were used in total. Insects were housed in colonies in an insectary at the University of Lincoln, that varied in temperature from 24 to 30 °C but had a 12/12 light/dark day cycle. They were fed ad libitum on a diet of bee pollen (Sevenhills, Wakefield, UK), fresh apple, and dry dog food (Pedigree Schmackos, UK), and had access to water through soaked cotton wool in a petri dish.

### Jumping apparatus

Jumping bush-crickets were filmed using a Photron FastCam Mini placed orthogonally to a small wooden platform. The camera filmed at a rate of 1000 frames per second. The bush-crickets were placed on the platform and orientated with their anterior–posterior axis aligned with the long side of the platform. This platform was placed at a fixed location, opposite a mock piece of wooden bark illuminated from above by a floodlight, and the surrounding exposed surfaces lined with black felt. This encouraged the bush-cricket to jump forward, towards the bark to ensure it remained in the focal plane of the camera during the entire video.

Bush-crickets were encouraged a jump by the experimenter by introducing quick movement (using the hands, pen, or paintbrush) to their visual field, i.e., roughly 100 degrees from its sagittal plane (Jeanrot et al. 1981), which elicits an anti-predator response (Burrows and Morris 2003). These jumps were only encouraged when the bush-cricket was positioned towards the edge of the platform nearest the bark, and jumps were only included in the dataset if all the bush-crickets’ legs were on the platform when initiating the jump. Additionally, if the bush-crickets jumped out of the camera's focal depth (either to the left or right of the platform) the jumps were discarded. In total, there were 269 recorded jumps from 67 individuals, with each bush-cricket jumping between 1 and 9 times, and an average of 4.0 (SD = 1.77) jumps per animal.

### Video analysis

Videos of the jumps were analysed using Tracker video analysis and modelling tool (Open Source physics, 2020). A scale was set using a ruler that was attached to the platform, which ensured that the distance between the scale and the locust was minimal and thus reducing the risk of lens distortion between the scale bar and the bush-cricket. Body length (BL) measurements (in mm) were taken using the Tracker software, starting at the most rostral point of the head and following the body along a straight line terminating at the most distal point of the abdomen. Length measurements (in mm) of the femur and tibia of the metathoracic leg were recorded and leg length (LL) was the combined length of the femur and tibia. Using the Tracker software, the head and centre of mass were recorded as x, y coordinates at three separate phases of the jump: the resting phase, the take-off phase, and ten frames after take-off (Fig. 1c, d). The coordinates at these points were used to calculate various parameters (see Table 1): the linear velocity (*v* in m/s); inertia (*I* in kg∙m^2^); translational kinetic energy (E_T_ in mJ); angular velocity ($\dot{\theta }$ in rads/s), rotational kinetic energy (E_R_ in mJ) and gravitational potential energy (GPE in mJ). Translational kinetic energy was calculated by summing E_T_ and GPE, thus accounting for the potential differences between bush-crickets of different sizes at take-off. The COM in locusts is located above the coxa of the hind leg (Bennet-Clark 1975) at the centre of the body (centre of rotation). The COM in the bush-cricket was thus chosen to be the same point, the centre of rotation, based on an understanding that the insect should function like a beam during the jump as described in Goode and Sutton (2023).

**Table 1:** Equations used to calculate parameters used in the analysis, where m = body mass (kg), d = displacement (m), t = time (s), g = gravitational field (9.8 m/s^2^), and h = difference in y position (height) between rest and take-off phase

Linear velocity (*v* in m/s)	$v= \sqrt{\left(\frac{{dx}^{2}}{dt}\right)+\left(\frac{{dy}^{2}}{dt}\right)}$	(1)
Inertia of a rod about its centre (*I* in kg $\cdot$ m^2^)	$I = \frac{1}{12}\left( {m \cdot {\text{body length}}^2 } \right)$	(2)
Translational kinetic energy (E_T_ in mJ)	${E}_{T}= \frac{1}{2}m \cdot {v}^{2}$	(3)
Angular velocity ($\dot{\theta }$ in rads/s)	$\dot{\theta }= \left(\frac{d\theta }{dt}\right)$	(4)
Rotational kinetic energy (E_R_ in mJ)	${E}_{R}= \frac{1}{2}I \cdot {\dot{\theta } }^{2}$	(5)
Gravitational potential energy (GPE in mJ)	$GPE=m\cdot g\cdot h$	(6)

### Statistical analyses

Data for each animal were averaged and Log_10_ transformed prior to analyses. Linear regressions were conducted to model the relationships between body mass (BM), body length (BL) and leg length (LL). Slopes generated within the regressions were compared against an expected isometric slope of 0.33 or 1.0 (Clemente and Dick 2023) using one-sample *t* tests (Bailey 1981). Scaling coefficients were calculated with a fit linear mixed-effects model (lmer; Kuznetsova et al. 2017) and computed within R studio (version 4.2.2, R Core development team 2022). This model allowed us to use all 269 as individual data points, while treating repeated measures for individual animals as a random factor. Finally, we tested for a difference in the mean proportions between the mean energy distributions between *S. gregaria* (*n* = 44) and *M. elongata* (*n* = 67). These data were not normally distributed so Wilcoxon rank sum test was conducted in R studio (version 4.2.2, R Core development team, 2022).

#### ***Hypotheses***

*Hypothesis one*: v increases proportionally with m (Hyp 1).

In muscle actuated jumpers, larger animals can generate greater take-off velocities (Sutton et al. 2019), which would predict a positive relationship between mass and linear velocity.

*Hypothesis two*: $\dot{\theta }$ decreases proportionally to m (Hyp 2).

Our second hypothesis predicted that there would be a negative scaling relationship between mass and angular velocity, with smaller bush-crickets experiencing a higher spin rate. Previous work on a latch mediated spring actuated system had $\dot{\theta }$ scaling with mass^−0.33^ (Goode and Sutton 2023) due to constant kinetic energy density across body sizes We wish to determine if the scaling for a muscle actuated system is different to the spring actuated systems given that their kinetic energy density should increase with muscle size (Sutton et al. 2019).

*Hypothesis three*: E_R_ is proportional to E_T_ (Hyp 3).

Finally, it was hypothesised that the bush-crickets would have a fixed rate of energy partitioning between translational and rotational kinetic energy, as observed in the locust (Goode and Sutton 2023).

## Results

### Scaling relationships of animal morphology

**Fig. 2:**
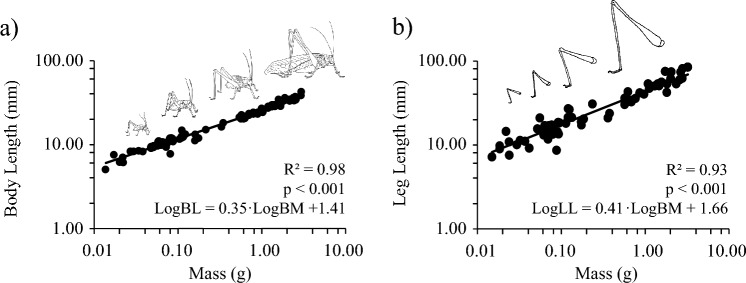
Scaling in *Mecopoda elongata*. **a** Relationship between body mass and body length. **b** Relationship between body mass and leg length. Under isometry, both relationships should scale with mass^0.33^

Body length ranged from 5.07 mm (0.014 g) to 42.12 mm (3.01 g) and showed a significant positive relationship with body mass: LogBL = 0.35·LogBM + 1.41 (*t*_slope_ = 64.19, *p* < 0.001, *R*^*2*^ = 0.98; Fig. 2a). The slope of this relationship (0.35, SE = 0.01) was significantly different from the predicted isometric value of 0.33 (*t*_65_ = 3.35, *p* = 0.002).

Leg length (LL, mm) also varied from 7.17 mm (0.014 g) to 84.47 mm (3.01 g) and significantly increased with body mass: LogLL = 0.41·LogBM + 1.66 (*t*_slope_ = 30.21, *p* < 0.001, *R*^2^ = 0.93; Fig. 2b). Like body length, the slope of this relationship (0.41, SE = 0.01) was significantly different from an isometric slope of 0.33 (*t*_65_ = 5.88 *p* < 0.001). Leg length also significantly increased with body length: LogLL = 1.17·LogBL + 0.005 (*t*_slope_ = 32.00, *p* < 0.001, *R*^2^ = 0.94) and exhibited significant positive allometry in relation to the expected isometric slope of 1.0 (*t*_65_ = 4.69, *p* < 0.001). The same scaling relationship was also observed for the tibia length: LogFL = 1.19·LogBL−0.0033 (*t*_slope_ = 29.22, *p* < 0.001, *R*^2^ = 0.93).

### Kinematics of the jump

**Fig. 3:**
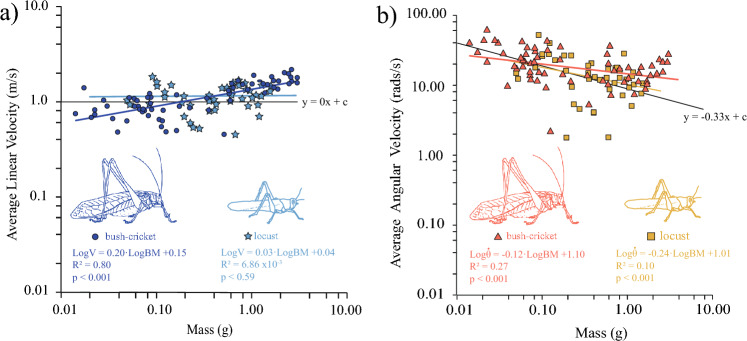
Relationship between body mass and take-off velocity during jumps by bush-crickets (*N* = 67) and locusts (*N* = 44). **a** Linear velocity (m/s) at take-off. **b** angular velocity (rads/s) at take-off. Locust data from Goode and Sutton (2023)

Across all jumps for the 67 bush-crickets, the average linear velocity ranged from 0.45 to 2.15 m/s. Linear velocity exhibited a significant positive relationship with increasing body mass (Fig. 3): Log*v* = 0.20·LogBM + 0.14 (*R*^*2*^ = 0.80), indicating that larger bush-crickets jumped faster. The slope of this relationship was significantly different from a slope of zero (*t*_267_ = 11.12, *p* < 0.001).

Angular velocity ($\dot{\theta }$) at take-off significantly decreased with increasing body mass: Log$\dot{\theta }$ = − 0.12·LogBM + 1.10 (*t*_slope_ = − 2.49, *p* < 0.001, *R*^2^ = 0.27; Fig. 3b), indicating smaller bush-crickets spun more quickly than larger bush-crickets (see Supplemental Movie 1). The slope of the observed relationship was significantly different from the predicted slope of − 0.33 (*t*_267_ = 4.16, *p* < 0.001).

### Energetics of the jump

**Fig. 4:**
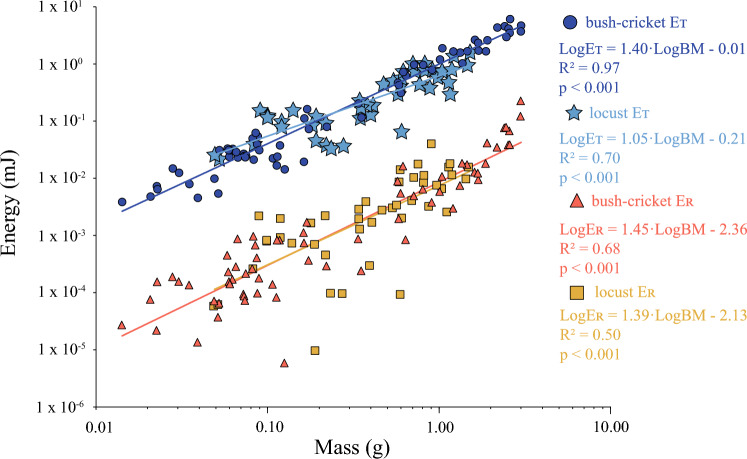
Translational kinetic energy (E_T_) and rotational kinetic energy (E_R_) of bush-cricket and locust jumps during take-off. Locust data from Goode and Sutton (2023)

The average translational kinetic energy of the bush-crickets jump at take-off increased proportionally with mass (Fig. 4). Average translational kinetic energy for the smallest bush-cricket (0.014 g) was 3.85 × 10^–3^ mJ compared with 4.67 mJ for the largest bush-cricket (3.01 g). Across all jumps for the 67 bush-crickets the average translational kinetic energy ranged from 3.85 × 10^–3^ mJ to 6.09 mJ. Translational kinetic energy varied significantly with mass: LogE_T_ = 1.40·LogBM− 0.013 (*t*_slope_ = 38.98, *p* < 0.001, *R*^2^ = 0.97). The slope of this relationship was significantly different from a slope of 1.0 (*t*_267_ = 11.12, *p* < 0.001).

Rotational kinetic energy (E_R_, mJ) at take-off increased with mass (Fig. 4), with the average rotational kinetic energy for the smallest bush-cricket (0.014 g) being 2.71 × 10^–5^ mJ. The average E_R_ for the largest bush-cricket (3.01 g) was 2.25 × 10^–1^ mJ and for all jumps E_R_ ranged from 5.89 × 10^–6^ to 2.25 × 10^–1^ mJ. Rotational kinetic energy varied significantly with mass: LogE_R_ = 1.45·LogBM− 2.36 (*t*_slope_ = 14.45, *p* < 0.001, *R*^2^ = 0.68). This slope was statistically different a slope of 1.0 (*t*_267_ = 4.43 *p* < 0.001). The slope for rotational kinetic energy (1.45) was not significantly different than the measured slope for translational kinetic energy (1.4) (*t*_267_ = 0.83, *p* = 0.28).

In the locust, the ratio of rotation and kinetic energy was constant independent of mass. Moreover, the kinetic energy density was also constant with size, which had the consequence that angular velocity had to scale with mass^−0.33^ to maintain this constant ratio (Goode and Sutton 2023). In the bush-cricket, kinetic energy varied significantly with size (Fig. 4), so it was possible to test if the ratio of rotational to translational kinetic energy was constant for bush-crickets of all sizes.

For the cricket rotational kinetic energy (${E}_{R}=\frac{1}{2} I{\dot{\theta }}^{2})$ divided by translational kinetic ${(E}_{T}=\frac{1}{2} m{v}^{2})$ is equal to $({L}^{2}\cdot {\dot{\theta }}^{2})/{v}^{2}$. Body length (L) was proportional to mass^0.35^ (Fig. 2a), linear velocity (v) was proportional to mass^0.20^ (Fig. 3a). Consequently if $({L}^{2}\cdot {\dot{\theta }}^{2})/{v}^{2}$ were constant, this simplifies down to $\frac{{E}_{R}}{{E}_{T}}=\frac{{({m}^{0.35})}^{2}\cdot {\dot{\theta }}^{2}}{{({m}^{0.20})}^{2}}$, therefore, a constant ratio between rotational kinetic energy and translational kinetic energy predicts that $\dot{\theta }\sim {m}^{-0.15}$. The observed slope with the hypothesised relationship was − 0.12 (Fig. 3b), which was not significantly different from a predicted slope of − 0.15 (*t*_267_ = 0.53, *p* = 0.35).

**Fig. 5:**
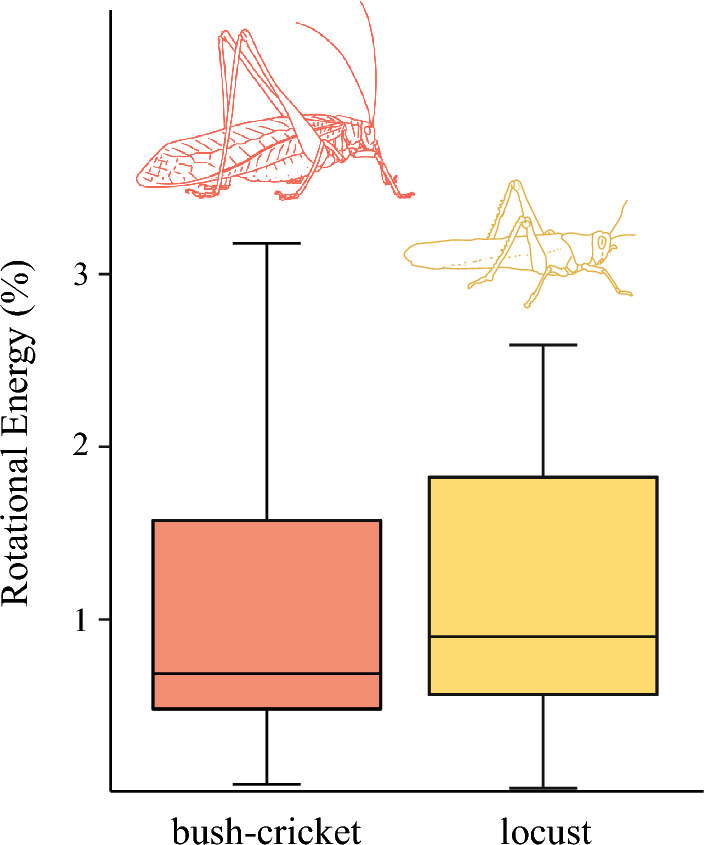
The percentage of total energy formed during take-off that goes into rotation for bush-crickets (*n* = 67) and locusts (*n* = 44). Locust data from Goode and Sutton (2023)

For bush-crickets, translational kinetic energy (scaling with mass^1.40^) and rotational kinetic energy (scaling with mass^1.45^), did not have significantly different relationships with animal mass. These results suggested that translational kinetic energy and rotational kinetic energy scaled proportionally with one another as mass increased. Therefore, it was possible to calculate an energy distribution for the bush-cricket jump, with the ratio of kinetic energy and rotational energy being constant and independent of the animal’s mass. On average 98.8% of the total energy distribution was used on producing linear velocity (including gravitational potential energy), while only 1.2% was used generating angular velocity (Fig. 5). The proportion of the energy distribution for generating angular velocity in bush-crickets was not statistically different from that of the locust (*W* = 1305, *n*_*1*_ = 44, *n*_*2*_ = 67, *p* = 0.310).

## Discussion

Here, we investigated rotation and energetics of muscle actuated (MA) jumps in the bush-cricket *Mecopoda elongata*. As predicted by Hyp 1, we found that larger bush-crickets can generate greater take-off velocities with a positive relationship between mass and linear velocity. This is due to a positive allometry between mass and leg length resulting in longer muscle actuation periods. Hyp 2, which predicted that smaller bush-crickets would experience more spin due to increased angular velocity, was also supported; a consequence of a reduced distance between the centre of mass (COM) and the head in smaller insects and an increased efficacy in transforming energy to vertical velocity (Scholz et al. 2005), making spin adjustment (overcoming the inertial forces generated during take-off) more challenging (Ilton et al. 2018). However, this angular velocity is lower than in similarly sized locusts due to a smaller velocity relative to body size in MA systems than LaMSA systems. We suggest that a combination of both factors influence spin in smaller animals. Finally, Hyp 3 predicted that the energy distribution formed during take-off would be fixed regardless of mass, and this was supported by the exponents between mass and the two measured kinetic energies (translational and rotational). When comparing the results to a mirrored study in the locust, we observed that smaller locusts outperformed bush-crickets in linear velocity because their power density was independent of mass (Goode and Sutton 2023). By contrast, bush-crickets had a greater linear velocity with increasing mass, which allowed larger bush-crickets to jump with a higher velocity than equally sized locusts. However, other published data by Gabriel (1985a) on jump velocity in adult locusts demonstrates velocities exceeding those discussed in (Goode and Sutton 2023), which result from morphological changes that occur between the 4th instar and adult phase of the locust (Gabriel 1985b). These morphological changes allow the adult locusts to generate jumps that are much faster than the 4th instar and younger locusts. It would be interesting to determine if extremely fast jumping adult locusts (with take-off velocities as high as 3–3.3 m/s) have the same energy distribution (99% kinetic energy, 1% rotational energy) as the evaluated bush-crickets (presented here) and the locusts from Goode and Sutton (2023). Adult locusts in the solitary phase of their life cycle (Rogers et al. 2016) can jump with even higher velocities (as high as 3.8 m/s) than the gregarious locusts observed in Gabriel (1985a, b), leaving another question open: do the solitarious adults spin faster when jumping with higher speeds to maintain the same energy distribution as bush-crickets and gregarious locusts?

In *M. elongata*, a greater linear velocity with increasing body mass may be important during shifts in predation ecology during life history. Adult *M. elongata* are regularly predated by substrate gleaning bats, and so defensive jumping and kicking behaviours may aid in predator escape (Prakash et al. 2021). Conversely, in the case of *S. gregaria* it is likely that the ability to perform powerful jumps as an adult is of less ecological value than the ability to jump with a consistent velocity across all instars, explained simply through considering their migratory behaviour (Kennedy 1951). Consistent linear velocity with mass may be a consequence of the LaMSA system, but selected for in this system as it increases efficiency, another element of jumping behaviour crucial for a migratory insect. This idea is supported by Goode and Sutton (2023), where it was found that a single locust was able to jump 44 times consecutively without experiencing a decline in linear velocity. However, many insects with LaMSA systems, such as planthoppers (Hemiptera: *Issus coleoptratus*) and fleas (Siphonaptera: *Archaeopsyllus erinacei*) are not migratory (Burrows 2010; Sutton and Burrows 2011), and thus the spring actuated system may serve other benefits in different ecological contexts.

In relation to the angular velocity between the two jumping systems, we observed that smaller bush-crickets spun slower than equally sized locusts. Controlling angular velocity may be more important for bush-crickets as they inhabit complex forest environments whereby navigation for mate attraction, resource acquisition, and predator avoidance will depend greatly on locomotion between a variety of substrates, across multiple planes of the habitat. Thus, jump accuracy, aided by reduced spin during take-off, may be an advantage of the MA system (Burrows et al. 2015; Sutton et al. 2016). In larger bush-crickets, however, spin during take-off was faster than equally sized locusts, and this was a consequence of the increased jump actuation from longer legs time having a greater effect on rotation around the fixed COM. After take-off, this can be adjusted to aid in landing or to initiate flight behaviour (Burrows et al. 2015).

Although larger bush-crickets may spin faster, they showed less than half the variation in spin rate between the largest and smallest individuals compared to the locust, supporting the notion that controlling angular velocity may be more important for bush-crickets across all instars. We suspect that this may be because the arboreal habits of many bush-crickets, where controlling movement across complex vegetation using finely controlled muscles will be more beneficial than a spring actuated system. Grasshoppers on the other hand tend to inhabit more open and/or terrestrial environments whereby movement through the environment is dominated by locomotion across the horizontal/azimuth plane (Ellis 1951). In this kind of locomotive system, the endurance offered by a LAMSA system may be more important for general locomotive behaviours (Gabriel 1985a, b) than the accuracy provided by MA systems. We hypothesise that control of spin in jumping insects offered by muscle actuation may be at a trade-off with the mass-independent velocity and long-distance efficiency of spring actuation.

Overall, in both groups, angular velocity decreased with increasing mass, supporting the idea that smaller insects spin more during a jump (Goode and Sutton 2023). This may be advantageous to younger insects as increased spin will result in a motion dazzle or flicker-fusion effect whereby the contour (outline) of the body becomes more challenging to identify, causing predators to misjudge prey direction (Hughes et al. 2014) or locate the prey at all against a complex background (Stevens 2007). While small insects can direct their jumps prior to take-off by manipulating yaw (Sutton and Burrows 2010), pitch is more challenging to control, but this unpredictable rotation may be beneficial. For bush-crickets and locusts around 0.2 g body mass, angular and linear velocity during take-off were comparable. It would be interesting to investigate the muscle architecture and power output of the jumping legs of the two systems at this mass to identify the differences and similarities within the two systems.

Despite differences in angular and linear velocities between the two orthopteran taxa, the pattern of the energy distributions of the jump were not significantly different. If this energy distribution is conserved across these two jumping mechanisms, why do the two different systems exist? One reason may be that larger muscle-driven jumpers can jump higher without springs (Sutton et al. 2016). Additionally, it may be found in the phase of the jump after take-off, or fine-tuning last minute decisions making during take-off to aid in accurate landing behaviour where the target is not stationary (e.g., branches, leaves, prey). This hypothesis is supported by studies of jumping mechanics in the mantis (*Stagmomantis theophila*; Mantodea, Mantidae), whereby manipulations of pitch after take-off permit accurate landing behaviour (Burrows et al. 2015) and are a function of mass and leg length (Sutton et al. 2016). In mantids, leg length scales isometrically (mass^0.33^), and linear velocity scales with mass^0.12^. In *M. elongata* on the other hand, length did not scale isometrically with mass, instead scaling to m^0.41^. This increase in leg length resulted in a longer contact time between the tarsi of the hindlegs and the substrate, increasing muscle work during take-off (Bobbert 2013) and proportionally increasing linear velocity as the animal gets larger (mass^0.20^). However, this increase in leg length should increase the acceleration period, increasing energy loss due to deformation of compliant surfaces, such as branches or leaves. In this experiment, where the substrate was solid, less energy should be lost to surface compliance. This proportional relationship suggests that there could be an underlying association between body mass, leg length and linear velocity in muscle actuated jumpers. In hemimetabolous insects, such as the Orthoptera and Mantodea, future identification of a length measure which confidently scales isometrically with mass could thus be combined with hind leg length measurements to predict exponents between mass and linear velocity in extinct taxa based on linear measurements of fossils.

Locusts (Caelifera) and bush-crickets (Ensifera) separated in the late Devonian (~ 360 million years ago; Song et al. 2015). Despite having very large differences in leg morphology, actuation mechanism, and life history, the two insects demonstrate a conserved partitioning of the energy distribution formed during take-off, with both having a size independent partitioning of about 99% linear kinetic energy and 1% rotational kinetic energy. Following take-off, however, muscle actuated jumpers can manipulate their spin for accurate landing (Burrows et al. 2015), something that has not been observed for the spring-actuated jumpers (Goode and Sutton 2023; Gvirsman et al. 2016; Cofer et al 2007), and a likely explanation for the evolution of the two different systems.

### Supplementary Information

Below is the link to the electronic supplementary material.

Supplementary file1 (DOCX 23 KB)

Supplementary file2 (MP4 15241 KB)

## Data Availability

All data is presented within the main text or supplemental material. High speed videos used in analysis are available upon request from GP Sutton.
