# Supplementary material for: Control of high-speed jumps in muscle and spring actuated systems: a comparative study of take-off energetics in bush-crickets (Mecopoda elongata) and locusts (Schistocerca gregaria)
Source: J Comp Physiol B. 2023 Oct 19;193(6):597–605. doi: 10.1007/s00360-023-01524-2 (PMC10613148; doi:10.1007/s00360-023-01524-2)
Supplement: Supplementary file 1 — Supplementary file1 (DOCX 23 KB) [file 360_2023_1524_MOESM1_ESM.docx]

**Supplemental information**

**Table S1**. Exponents and intercepts from all linear models (N=67, p < 0.001 for all models tested)

| Model | Exponent (SE) | T-value | R^2^ |
| --- | --- | --- | --- |
| **Log body length * Log body mass** | | | |
| Intercept | 1.41 (±0.005) | 281.44 | 0.98 |
| Slope | 0.35 (±0.005) | 64.19 |  |
| **Log leg length * Log body mass** | | | |
| Intercept | 1.66 (±0.013) | 132.35 | 0.93 |
| Slope | 0.41 (±0.014) | 30.21 |  |
| **Log femur length * Log body length** |  |  |  |
| Intercept | -0.033 (±0.049) | -6.64 | 0.93 |
| Slope | 1.19 (±0.041) | 29.22 |  |
| **Log leg length * Log body length** | | | |
| Intercept | 0.005 (±0.045) | 0.10 | 0.94 |
| Slope | 1.17 (±0.037) | 32.00 |  |

**Table S2**. Exponents and intercepts from all lmer models (N=269, p <0.01 for all models tested).

| Model | Exponent (SE) | T-value | R^2^ |
| --- | --- | --- | --- |
| **Log linear velocity * Log body mass** | | | |
| Intercept | 0.14 (±0.016) | 8.84 | 0.80 |
| Slope | 0.20 (±0.018) | 11.12 |  |
| **Log angular velocity * Log body mass** | | | |
| Intercept | 1.10 (±0.043) | 25.284 | 0.27 |
| Slope | -0.12 (±0.048) | -2.49 |  |
| **Log translational kinetic energy * Log body mass** | | | |
| Intercept | -0.01 (±0.033) | -0.41 | 0.97 |
| Slope | 1.40 (±0.036) | 38.98 |  |
| **Log rotational kinetic energy * Log body mass** | | | |
| Intercept | -2.34 (±0.088) | -26.51 | 0.64 |
| Slope | 1.44 (0.099) | 14.45 |  |

**Movie S1.** Take-off phase of jumping in a first instar and adult of *Mecopoda elongata*.
